# A Novel Stochastic Approach for Static Damage Identification of Beam Structures Using Homotopy Analysis Algorithm

**DOI:** 10.3390/s21072366

**Published:** 2021-03-29

**Authors:** Zhifeng Wu, Bin Huang, Kong Fah Tee, Weidong Zhang

**Affiliations:** 1 School of Civil Engineering and Architecture, Wuhan University of Technology, Wuhan 430070, China; wuzhifeng_tujian@whut.edu.cn (Z.W.); whlqfy@163.com (W.Z.); 2 School of Engineering, University of Greenwich, Kent ME4 4TB, UK; K.F.Tee@gre.ac.uk

**Keywords:** stochastic damage identification, modelling error, measurement error, homotopy analysis algorithm, static condensation, L1 regularization

## Abstract

This paper proposes a new damage identification approach for beam structures with stochastic parameters based on uncertain static measurement data. This new approach considers not only the static measurement errors, but also the modelling error of the initial beam structures as random quantities, and can also address static damage identification problems with relatively large uncertainties. First, the stochastic damage identification equations with respect to the damage indexes were established. On this basis, a new homotopy analysis algorithm was used to solve the stochastic damage identification equations. During the process of solution, a static condensation technique and a L1 regularization method were employed to address the limited measurement data and ill-posed problems, respectively. Furthermore, the definition of damage probability index is presented to evaluate the possibility of existing damages. The results of two numerical examples show that the accuracy and efficiency of the proposed damage identification approach are good. In comparison to the first-order perturbation method, the proposed method can ensure better accuracy in damage identification with relatively large measurement errors and modelling error. Finally, according to the static tests of a simply supported concrete beam, the proposed method successfully identified the damage of the beam.

## 1. Introduction

In recent decades, the construction of bridges has developed rapidly in China. Due to the imperfection of construction or long periods of operation, bridge structures usually have some degree of damage. Similar to the bridge structures, many beam structures in civil engineering face the same safety problem. Therefore, the damage identification of beam structures including bridges has attracted increasing attention of researchers [1,2,3,4,5,6,7,8,9,10,11,12,13,14,15].

For most damage identification techniques, dynamic measurement data are often employed [16]. Generally, dynamic tests can provide more information and are easier to implement than static tests during the service life of structures. However, for some certain types of structures, such as beam structures, static tests can be performed as easily as dynamic tests. For the static identification methods, the test equipment required are relatively cheap and the static displacements or strains of structures can be measured accurately. In addition to boundary conditions, the dynamic damage identification is affected by not only structural stiffness but also mass and damping ratio. However, for static identification, only structural stiffness needs to be considered.

In view of this, the static damage identification of beam structures is paid more attention. Three types of static damage identification methods have been proposed, which are based on the measured static displacements [1,2,3,17,18,19], static strains [4,5,20], and both the static displacements and strains [6]. For example, Lakshmanan [1] applied a least square error-based solution method for the estimation of flexural rigidities and damages of beam structures by the static measured deformation values. Lu [2] used the incomplete static measured displacement to localize the damage in the element level of the beam structures. Guo [3] presented a novel static approach to identify the damage of beams based on the minimum constitutive relation error. Unlike straight beams, Greco [17] dealt with the inverse problem of damage identification in an elastic parabolic arch using the static displacements. Based on static strain measurements, Shenton [4] proposed a damage identification procedure using a genetic algorithm in a fixed-fixed beam structure. Liu [5] introduced a Brillouin optical time domain analysis technique integrated with quasi-static strain influence lines to locate the damage of girders. Combining the strain with displacement, Maity [6] used static information as possible candidates of a back-propagation neural network damage identification approach to detect existing damages of a simple cantilever beam. Not restricted to the beam structures, Yang [18] made use of the new flexibility disassembly technique based on the static responses for the damage localization and quantification of truss structures. Rezaiee-Pajand [19] presented a new algorithm for static damage detection of two- and three-dimensional frames based on static displacements. Seyedpoor [20] used the static strain energy as an index to identify damage locations of the truss and frame structures.

To acquire more available data, some researchers also used identification algorithms based on the combination to dynamic and static test data. Wang [21] presented a two-stage identification algorithm to identify the structural damage by employing the changes in natural frequencies and static measured displacements. Raghuprasad [22] achieved damage identification through static deflection values and dynamic modal frequencies. Lu [23] tried to identify the damage using noisy natural frequency and displacement measurements. Yang [24] proposed a new combined static–dynamic method for structural damage identification. However, in the literature [23,24], it was obviously found that the statically identified results appear to be different to those from the dynamic estimates. This problem deserves further study.

In summary, the above methods are able to use static measurement data to identify structural damage assuming that the structural modelling is certain. Compared with the damage identification of deterministic structures, structural damage identification methods based on the theory of probability can reflect the uncertain nature of the damage problems with stochastic parameters and measurement error or noise. It is expected to realize the damage identification of engineering structures statistically by solving random inverse problems [25]. Regarding this aspect, some researchers have conducted many active works. For example, Caddemi [26,27] used a Monte Carlo method to simulate the noise which was modelled as a random variable and presented explicit solutions of the damage identification problem for the case of simply supported beams. Wang [28] achieved static flexibility measurement by adding a Gauss noise to one sample of the real measured flexibilities, and then identified the boundary conditions of the tapered beam-like structures. Compared with the literature [4], Hu [29] investigated the effect of measurement error, which is modelled as a Gaussian, zero-mean, random variable, using Monte Carlo simulation. However, the effect of the uncertain modelling error on the damage identification results was not investigated. For finite element-based static identification methods, the literature seldom statistically considers the effect of both the measurement error and the modelling error on the damage estimation of structures. Therefore, it is important to develop new static data-based damage methods to address the uncertainty errors in measurement and modelling.

At present, a number of stochastic static damage identification methods have been developed, including the Monte Carlo simulation methods [26,27,28,29] and the perturbation methods [30,31,32,33]. Due to the versatility of Monte Carlo simulation, it is commonly used to investigate the random characteristics of identified results and verify the accuracy of other stochastic identification methods. However, the computational power and workload of applying Monte Carlo simulation to large-scale complex problems are demanding. The perturbation method is another commonly used and efficient tool to address the uncertainty in stochastic damage identification. For example, Yu [30] applied the first-order perturbation method to detect small structural damage of a cantilever composite plate with a single crack. Yin [31] proposed a statistical damage detection approach based on the dynamical model reduction technique and the perturbation technique. He [32] used the first-order and second-order perturbation equations of curvature modal shape and frequency to identify the damage. Wong [33] iteratively used the perturbation method in conjunction with an optimization method to identify the stiffness parameters of damaged structures. In these works, the efficiency of these perturbation methods was high, but they were limited to small uncertainties of damage. When addressing relatively large damage, it cannot always ensure the accuracy of damage identification.

Thus, following the idea of finite element-based stochastic damage identification methods, a homotopy analysis algorithm is introduced to address the stochastic static identification problems with relatively large uncertainty of damage. Based on the concept of homotopy, this paper focuses on a novel stochastic approach for the damage identification of beam structures using static measurement data. The new static damage identification method considers not only the initial modelling errors, but also the static measurement errors. The initial models of beam structures are regarded as random because the modelling error is inevitable. In addition, the measurement errors are assumed to be uncertain. After the stochastic identification equations for damage indexes are established, the homotopy analysis algorithm is used to solve the identification equations. Because the measured degrees of freedom (DOFs) of the identified beam structures are usually limited, some of them are unavailable, and a static condensation technique is employed to solve the stochastic damage identification. To address the ill-posed problems caused by incomplete measurement information and static measurement errors, the L1 regularization method is used to solve the homotopy damage equations. Additionally, the damage probability indexes of beam structures are defined. Two numerical examples, a simply supported beam and a continuous beam, are provided to demonstrate the identification effectiveness of the proposed method. In the damage identification of the continuous beam with various cross-sections, the first-order perturbation method is used to compare with the proposed method. Furthermore, a series of static tests of a simply supported concrete beam is implemented to verify the new stochastic identification method. The numerical and experimental results indicate that the proposed new stochastic identification method can identify structural damage effectively and quickly. It is also expected that this new method can be applied to the damage detection of other types of structures, such as various smart structures.

## 2. Theory

### 2.1. Stochastic Static Damage Identification Equation

For a beam structural system with *N* degrees of freedom, the static equilibrium equations of the structure at the initial intact state and damaged state can be expressed, respectively, as:

(1) $$K_{0}x_{0}=F_{0}$$

(2) $$K_{d}x_{d}=F_{d}$$

where $K_{0}$ and $K_{d}$ are *N* × *N* dimensional stiffness matrixes at the initial intact state and damaged state, respectively. $x_{0}$ and $x_{d}$ denote the displacement vectors of the structure at the initial and damaged states, respectively. $F_{0}$ and $F_{d}$ are static load vectors related to the initial and damaged states, respectively.

It is assumed that the damage of the beam structure derives from a change of elastic modulus or bending rigidity of the structure at the element level, and this change will lead to the variation, ΔK, of the stiffness matrix of the initial structure, which is represented by:

(3) $$\Delta K=\sum_{i=1}^{n}\alpha_{i}K_{i}$$

where *n* is the total number of beam structural elements, $\alpha_{i}$ is the damage index of the *i*-th element, which means the variation of elastic modulus or bending rigidity of the *i*-th element. $K_{i}$ is a *N* × *N* dimensional expanded matrix of the *i*-th element stiffness matrix of the beam structure, where all elements in the expanded parts are zeros.

Because the damage of the beam structure causes the change of the structural stiffness, the stiffness matrix $K_{d}$ and the displacement vector $x_{d}$ of the damaged beam structure can be expressed, respectively, as:

(4) $$K_{d}=K_{0}+\Delta K$$

(5) $$x_{d}=x_{0}-\Delta x$$

Substituting Equation (3) into Equation (4), one can obtain:

(6) $$K_{d}=K_{0}+\sum_{i=1}^{n}\alpha_{i}K_{i}$$

Considering that the loads at the initial state and the damaged state are identical, the following equation can be easily obtained:

(7) $$K_{0}x_{0}=K_{d}x_{d}=F_{0}$$

Substituting Equation (6) into Equation (7) yields:

(8) $$K_{0}x_{0}=(K_{0}+\sum_{i=1}^{n}\alpha_{i}K_{i})x_{d}$$

Then, Equation (8) can be rewritten as:

(9) $$\sum_{i=1}^{n}\alpha_{i}K_{i}x_{d}=K_{0}\Delta x$$

where $\Delta x=x_{0}-x_{d}$.

Here, Equation (9) is the deterministic static damage identification equation with respect to the element damage indexes $\alpha_{i}\;\left(i=1,\ldots ,n\right)$.

To identify the damage of a beam structure, it is necessary to establish the initial model of the structure at its intact status. However, compared with the true structure, the modelling error of the initial model is unavoidable. Here, this modelling error refers to the uncertainty of structural parameters. Because the practical structural parameters are bounded regardless of whether they are material or geometrical, the structural parameters can be assumed to be random variables $\xi_{j}(j=1,2,\cdots ,m)$ with beta distributions. *m* is the number of stochastic variables corresponding to the initial modelling error. Clearly, for the proposed method, the structural parameters can be of any distribution, such as the lognormal distribution, according to actual situations. Under the above assumption, the stiffness matrix $K_{0}$ and the displacement vector $x_{0}$ of the intact structure can be expressed as the functions of the stochastic variables $\xi_{j}(j=1,2,\cdots ,m)$, respectively, as:

(10) $$K_{0}=K_{0a}+\sum_{j=1}^{m}\xi_{j}K_{0j}$$

(11) $$x_{0}=x_{0a}+\sum_{j=1}^{m}\xi_{j}x_{0j}$$

where $K_{0a}$ and $x_{0a}$ are the mean of the stiffness matrix $K_{0}$ and the displacement vector $x_{0}$, respectively. $\sum_{j=1}^{m}\xi_{j}K_{0j}$ and $\sum_{j=1}^{m}\xi_{j}x_{0j}$ are the uncertain parts of $K_{0}$ and $x_{0}$, respectively. $K_{0j}$ and $x_{0j}$ are the adjoint parts of $K_{0}$ and $x_{0}$ with respect to $\xi_{j}$, respectively.

The stochastic variables are assumed to be completely dependent, which means that $\xi_{j}=\xi (j=1,2,\cdots ,m)$. Assume that $K_{0b}=[K_{01},K_{02},\cdots ,K_{0m}]$ and $x_{0b}=[x_{01},x_{02},\cdots ,x_{0m}]$. Then, considering that the damage index $\alpha_{i}$ is the function of the stochastic variable ξ, one can rewrite Equation (8) as:

(12) $$\left(K_{0a}+\xi K_{0b}\right)\left(x_{0a}+\xi x_{0b}\right)=\left[\left(K_{0a}+\xi K_{0b}\right)+\sum_{i=1}^{n}\alpha_{i}\left(\xi \right)K_{i}\right]x_{d}$$

The obtained Equation (12) is the stochastic damage identification equation with the stochastic variable ξ related to the initial modelling error.

In practice, the measured static data will also result in the uncertainty of the damage identification. The static measurement error and the initial modelling error are independent. The measurement errors of the static data can be described by another independent random variable η, which can be modelled as a beta distribution because the measurement errors are bounded.

If the displacements of the damaged structure are measured, it can be represented as:

(13) $$x_{d}=x_{da}+\xi x_{db}$$

where $x_{da}$ denotes the deterministic part of the measured displacement vector $x_{d}$ of the damaged structure and $\eta x_{db}$ is the stochastic part of $x_{d}$. $x_{db}$ is the adjoint vector for the random variable η.

Here, the damage index $\alpha_{i}$ becomes the joint function of the stochastic variable ξ and η. Substituting Equation (13) into Equation (12) leads to:

(14) $$\left(K_{0a}+\xi K_{0b}\right)\left(x_{0a}+\xi x_{0b}\right)=\left[\left(K_{0a}+\xi K_{0b}\right)+\sum_{i=1}^{n}\alpha_{i}\left(\xi ,\eta \right)K_{i}\right]\left(x_{da}+\eta x_{db}\right)$$

Hence, the stochastic damage identification equation in Equation (14) is achieved considering the uncertainty of the initial modelling error and the static measurement errors.

### 2.2. Homotopy Solution of the Stochastic Damage Identification Equation

Many stochastic finite element methods [34,35,36,37,38,39,40], such as the generalized spectral stochastic finite element method [36] and stochastic reduced basis methods [37], may be used to solve Equation (14). For a balance between efficiency and accuracy, it is recommended in this paper to utilize a new proposed homotopy analysis algorithm to determine the solution of the stochastic damage identification equation in Equation (14). The homotopy analysis algorithm in the literature [38,39,40] can be used to establish a homotopy relationship between the deterministic damage index and the stochastic damage index.

For convenience of homotopy analysis, Equation (14) can be rewritten as:

(15) $$(S_{0}+\eta S_{1})\alpha =K_{0a}x_{0a}-T_{0}+\xi \left(T_{1}+T_{2}-T_{3}\right)+\xi^{2}T_{4}-\xi \eta T_{5}-\eta T_{6}$$

where $\alpha =[\alpha_{1},\alpha_{2},\cdots ,\alpha_{n}]$, $S_{0}=[K_{1}x_{da},K_{2}x_{da},\ldots ,K_{n}x_{da}]$, $S_{1}=[K_{1}x_{db},K_{2}x_{db},\ldots ,K_{n}x_{db}]$, $T_{0}=K_{0a}x_{da}$, $T_{1}=K_{0a}x_{0b}$, $T_{2}=K_{0b}x_{0a}$, $T_{3}=K_{0b}x_{da}$, $T_{4}=K_{0b}x_{0b}$, $T_{5}=K_{0b}x_{db}$, $T_{6}=K_{0a}x_{db}$ and $H_{0}=\xi \left(T_{1}+T_{2}-T_{3}\right)+\xi^{2}T_{4}$$-\xi \eta T_{5}-\eta T_{6}$.

Now one can reconstruct a new function Ω(ξ,η,h,p) of the stochastic variables ξ, η and the parameters h, p. When p=0, $\Omega (\xi ,\eta ,h,0)=\alpha_{0}$; when p=1, Ω(ξ,η,h,1)=α(ξ,η,h). $\alpha_{0}$ is the deterministic damage index vector in Equation (9), $\alpha_{0}=S_{0}^{-1}\left(K_{0a}x_{0a}-T_{0}\right)$, which can be solved from Equation (15).

Then, to construct the relationship between the initial approximation solution $\alpha_{0}$ and the stochastic damage index α(ξ,η,h) or the final stochastic solution of the damage index vector:

(16) $$\begin{array}{l} \left(1-p)(S_{0}\Omega (\xi ,\eta ,h,p)-S_{0}\alpha_{0}\right)\\ =ph[(S_{0}+\eta S_{1})\Omega (\xi ,\eta ,h,p)-(K_{0a}x_{0a}-T_{0}+H_{0})] \end{array}$$

where h is an auxiliary parameter and h≠0. Equation (16) is the zero-order deformation equation, which represents the homotopy relationship between $\alpha_{0}$ and α(ξ,η,h).

The Maclaurin series expansion of the function Ω(ξ,η,h,p) with respect to the parameter p can be represented as:

(17) $$\Omega (\xi ,\eta ,h,p)=\Omega (\xi ,\eta ,h,0)+\sum_{k=1}^{\infty }\left(\frac{\Omega^{\left[k\right]}(\xi ,\eta ,h,0)}{k!}\right)p^{k}$$

where *k* is the number of the order of the homotopy series expansion. $\Omega^{\left[k\right]}(\xi ,\eta ,h,0)$ means the k-th order partial derivative of Ω(ξ,η,h,p) with respect to p at p=0, which can be obtained by taking the k-th partial derivative of the zero-order deformation equation as illustrated in the following derivation.

The first-order partial derivative of Equation (16) with respect to p can be expressed as:

(18) $$\begin{array}{l} -[S_{0}\Omega -S_{0}\alpha_{0}]+(1-p)S_{0}\Omega^{\left[1\right]}\\ =h[(S_{0}+\eta S_{1})\Omega -\left(K_{0a}x_{0a}-T_{0}+H_{0}\right)]+ph(S_{0}+\eta S_{1})\Omega^{\left[1\right]} \end{array}$$

where Ω is the abbreviation of Ω(ξ,η,h,p). Letting p=0, Equation (18) can be written as:

(19) $$S_{0}\Omega_{}^{\left[1\right]}|{}_{p=0}=h[\left(S_{0}+\eta S_{1}\right)\alpha_{0}-\left(K_{0a}x_{0a}-T_{0}+H_{0}\right)]$$

Considering that $S_{0}\alpha_{0}-\left(K_{0a}x_{0a}-T_{0}\right)=0$, then:

(20) $$\Omega_{}^{\left[1\right]}|{}_{p=0}=\left(-h\right)\left(\eta \alpha_{1}+H_{1}\right)$$

where $\alpha_{1}=-S_{0}^{-1}S_{1}\alpha_{0}$, $H_{1}=S_{0}^{-1}H_{0}$. Then, $\Omega^{\left[1\right]}(\xi ,\eta ,h,0)$ can be obtained.

Taking the partial derivative of the first-order deformation equation in Equation (18) with respect to p yields:

(21) $$-2S_{0}\Omega_{}^{\left[1\right]}+(1-p)S_{0}\Omega_{}^{\left[2\right]}=2h(S_{0}+\eta S_{1})\Omega^{\left[1\right]}+ph(S_{0}+\eta S_{1})\Omega_{}^{\left[2\right]}$$

Equation (21) is the second-order deformation equation, according to which, letting p=0 leads to the second-order derivative of the function Ω(ξ,η,h,p) with respect to p as follows:

(22) $$\Omega_{}^{\left[2\right]}\;|{}_{p=0}=2\left(-h\right)\left(1+h\right)\left(\eta \alpha_{1}+H_{1}\right)+2{\left(-h\right)}^{2}\left(\eta^{2}\alpha_{2}+\eta H_{2}\right)$$

where $\alpha_{2}=-S_{0}^{-1}S_{1}\alpha_{1}$, $H_{2}=-S_{0}^{-1}S_{1}H_{1}$.

Similar to the above steps, the third- and fourth-order derivatives of the function Ω(ξ,η,h,p) with respect to p can be determined, respectively. When p=0:

(23) $$\begin{array}{l} \Omega_{}^{\left[3\right]}|{}_{p=0}=6(-h){\left(1+h\right)}^{2}\left(\eta \alpha_{1}+H_{1}\right)+12{\left(-h\right)}^{2}(1+h)\left(\eta^{2}\alpha_{2}+\eta H_{2}\right)\\ \;+6{\left(-h\right)}^{3}\left(\eta^{3}\alpha_{3}+\eta^{2}H_{3}\right) \end{array}$$

(24) $$\begin{array}{l} \Omega_{}^{\left[4\right]}{|}_{p=0}=24(-h){\left(1+h\right)}^{3}\left(\eta \alpha_{1}+H_{1}\right)+72{\left(-h\right)}^{2}{\left(1+h\right)}^{2}\left(\eta^{2}\alpha_{2}+\eta H_{2}\right)\\ \;+72{\left(-h\right)}^{3}(1+h)\left(\eta^{3}\alpha_{3}+\eta^{2}H_{3}\right)+24{\left(-h\right)}^{4}\left(\eta^{4}\alpha_{4}+\eta^{3}H_{4}\right) \end{array}$$

where $\alpha_{3}=-S_{0}^{-1}S_{1}\alpha_{2}$, $\alpha_{4}=-S_{0}^{-1}S_{1}\alpha_{4}$, $H_{3}=-S_{0}^{-1}S_{1}H_{2}$, $H_{4}=-S_{0}^{-1}S_{1}H_{3}$.

The other deformation equations $\Omega_{}^{\left[k\right]}|{}_{p=0}$ can be acquired in the same manner by taking the k-th partial derivative of the zero-order deformation equation with respect to the parameter p at p=0.

Substituting $\Omega_{}^{\left[k\right]}|{}_{p=0}$ into Equation (17), and letting p=1, the Maclaurin series expansion of the function Ω(ξ,η,h,p) can be expressed as:

(25) $$\alpha \left(\xi ,\eta ,h\right)=\Omega (\xi ,\eta ,h,1)=\alpha_{0}+\beta_{k,1}\left(h\right)\lambda_{1}+\beta_{k,2}\left(h\right)\lambda_{2}+\cdots +\beta_{k,l}\left(h\right)\lambda_{l}+\cdots$$

where $\lambda_{l}=\eta^{l}\alpha_{l}+\eta^{l-1}H_{l}(l\geq 1)$, and $\beta_{k,l}\left(h\right)$(l=1,2,⋯,k) is the approaching approximate function and can be given as follows:

(26) $$\beta_{k,l}(h)=\{\begin{array}{c} 0\;\;\;\;\;\;\;\;\;(t>k)\\ {\left(-h\right)}^{t}\sum_{l=0}^{k-t}\left(\begin{array}{c} l+t-1\\ l \end{array}\right){\left(1+h\right)}^{l}\;(1\leq t\leq k)\\ 1\;\;\;\;\;\;\;\;\;(t<0) \end{array}$$

In regard to the approaching function $\beta_{k,l}\left(h\right)$, the homotopy parameter *h* can control the convergence range and speed of the homotopy series in Equation (25), which shows the superiority of the homotopy series over the traditional Taylor series. The limited order terms are selected to improve the calculation efficiency in the damage identification procedure. $\alpha_{\left(k\right)}(\xi ,\eta ,h)$ can be used to represent the k-th order approximate solution of the homotopy series. For the damage index of the *i*-th element, there is an inevitable approximation error between the homotopy series solution $\alpha_{i}(\xi ,\eta ,h)$ and the *k*-th order approximate solution $\alpha_{(k),i}(\xi ,\eta ,h)$; the relative error between them can be defined as:

(27) $$\Lambda_{i}\left(\xi ,\eta ,h\right)=\left|\frac{\alpha_{i}\left(\xi ,\eta ,h\right)-\alpha_{\left(k\right),i}\left(\xi ,\eta ,h\right)}{\alpha_{i}\left(\xi ,\eta ,h\right)}\right|\left(i=1,2,\cdots ,n\right)$$

where the homotopy parameter h can be achieved by minimizing the errors $\Lambda_{i}(\xi ,\eta ,h)$(i=1,2,⋯,n).

Assume that the mean and standard deviation of the stochastic parameter vector {ξ,η} are $\left\{\mu_{1},\mu_{2}\right\}$ and $\left\{\sigma_{1},\sigma_{2}\right\}$, respectively. Then one can assume that $\xi_{\varphi }=\mu_{1}+\varphi \sigma_{1}$ and $\eta_{\varphi }=\mu_{2}+\varphi \sigma_{2}$ (φ = 1, 2, 3), which are three samples in a sample space of the random variables ξ and η. The deterministic damage index $\alpha_{di}(\xi_{\varphi },\eta_{\varphi })$ of the *i*-th element with respect to the three samples can be obtained by solving Equation (14). These damage indexes are regarded as the exact solutions. Then, the *k*-th order homotopy approximate solutions $\alpha_{\left(k\right),i}(\xi_{\varphi },\eta_{\varphi },h)$(i=1,2,⋯,n; φ=1,2,3) of the damage index of the *i*-th element can be obtained, which only depends on the parameter h.

To obtain the optimal solution of the parameter h, the objective functions $W_{\varphi }\left(h\right)$ are defined as:

(28) $$\begin{array}{cc} W_{\varphi }\left(h\right)=\sum_{i=1}^{n}{\left(\alpha_{di}\left(\xi_{\varphi },\eta_{\varphi }\right)-\alpha_{\left(k\right),i}(\xi_{\varphi },\eta_{\varphi },h)\right)}^{2} & \end{array}\begin{array}{cc} \left(\varphi =1,2,3\right) & \end{array}$$

The appropriate value of h can be found by optimizing the objective functions $W_{\varphi }\left(h\right)$ so that the error functions $\Lambda_{i}(\xi ,\eta ,h)$(i=1,2,…,n) are as small as possible.

### 2.3. Static Condensation of Damage Identification

In actual damage identification of beam structures, only the responses at limited measurement points can be recorded. Therefore, a static condensation method [41] is used to eliminate unmeasured responses. First, rewriting the matrices in Equation (1) as the partitioned matrices, Equation (1) becomes:

(29) $$\left[\begin{array}{cc} K_{0vv} & K_{0v\theta }\\ K_{0\theta v} & K_{0\theta \theta } \end{array}\right]\left\{\begin{array}{c} x_{0v}\\ x_{0\theta } \end{array}\right\}=\left\{\begin{array}{c} F_{0v}\\ F_{0\theta } \end{array}\right\}$$

where v and θ denote the numbers of measured and unmeasured DOFs, respectively, and v+θ=N. $K_{0vv}$ and $K_{0v\theta }$ are the submatrices of $K_{0}$ with respect to the measured DOFs, respectively. $K_{0\theta v}$ and $K_{0\theta \theta }$ are the submatrices of $K_{0}$ corresponding to the unmeasured DOFs, respectively. $x_{0v}$ and $x_{0\theta }$ are the measured and unmeasured parts of $x_{0}$, respectively. $F_{0v}$ and $F_{0\theta }$ are the measured and unmeasured static force vectors, respectively. Considering that for an actual intact structure, only the vertical load is added, and the unmeasured force vector $F_{0\theta }$ equals zero, Equation (30) can be obtained by Equation (29), as:

(30) $$x_{0\theta }=-K_{0\theta \theta }^{-1}K_{0\theta v}x_{0v}$$

Substituting $x_{0\theta }$ in Equation (30) into Equation (29) yields:

(31) $$(K_{0vv}-K_{0v\theta }K_{0\theta \theta }^{-1}K_{0\theta v})x_{0v}=F_{0v}$$

Further, Equation (31) can be rewritten as:

(32) $$K_{0v}x_{0v}=F_{0v}$$

where $K_{0v}=K_{0vv}-K_{0v\theta }K_{0\theta \theta }^{-1}K_{0\theta v}$.

Similarly, by condensing the stiffness matrix $K_{d}$ in Equation (2), the submatrix $K_{dv}$ corresponding to $K_{0v}$ can be expressed as:

(33) $$K_{dv}=K_{dvv}-K_{dv\theta }K_{d\theta \theta }^{-1}K_{d\theta v}$$

where $K_{dvv}$ and $K_{dv\theta }$ are the submatrices of $K_{d}$ with respect to the measured DOFs, respectively. $K_{d\theta v}$ and $K_{d\theta \theta }$ are the submatrices of $K_{d}$ corresponding to the unmeasured DOFs, respectively.

Using Equations (6) and (33) can be rewritten as:

(34) $$K_{dv}=(K_{avv}+\sum_{i=1}^{n}\alpha_{i}K_{ivv})-(K_{av\theta }+\sum_{i=1}^{n}\alpha_{i}K_{iv\theta })(K_{a\theta \theta }+\sum_{i=1}^{n}\alpha_{i}K_{i\theta \theta }{)}^{-1}(K_{a\theta v}+\sum_{i=1}^{n}\alpha_{i}K_{i\theta v})$$

The partial derivative of Equation (34) with respect to $\alpha_{i}$ is expressed as:

(35) $${\frac{\partial K_{dv}}{\partial \alpha_{i}}|}_{\alpha_{i}=0}=K_{ivv}-K_{i\theta v}K_{0\theta \theta }^{-1}K_{0\theta v}-K_{0v\theta }K_{0\theta \theta }^{-1}K_{i\theta v}+K_{0v\theta }K_{0\theta \theta }^{-1}K_{i\theta \theta }K_{0\theta \theta }^{-1}K_{0\theta v}$$

Then, the first-order Taylor expansion of $K_{dv}$ at $\alpha_{i}=0$ can be written as:

(36) $$K_{dv}={K_{dv}|}_{\alpha_{i}=0}+\sum_{i=1}^{n}\alpha_{i}{\frac{\partial K_{dv}}{\partial \alpha_{i}}|}_{\alpha_{i}=0}$$

Substituting Equation (36) into Equation (7) yields:

(37) $$K_{0v}x_{0v}={(K_{dv}|}_{\alpha_{i}=0}+\sum_{i=1}^{n}\alpha_{i}{\frac{\partial K_{dv}}{\partial \alpha_{i}}|}_{\alpha_{i}=0})x_{dv}$$

When $\alpha_{i}=0$, it means that the stiffness of initial and damaged structure does not change, which is ${K_{dv}|}_{\alpha_{i}=0}=K_{0v}$. Assuming that $K_{vi}=\sum_{i=1}^{n}\alpha_{i}{\frac{\partial K_{dv}}{\partial \alpha_{i}}|}_{\alpha_{i}=0}$ and $\Delta x_{v}=x_{0v}-x_{dv}$, Equation (37) is rewritten as:

(38) $$\sum_{i=1}^{n}\alpha_{i}K_{vi}x_{dv}=K_{0v}\Delta x_{v}$$

The above Equation (38) is similar to Equation (9), and is the deterministic damage identification equation for the damage index after static condensation.

When considering the uncertainty of the initial modelling error and the static measurement errors, Equation (38) can be rewritten as:

(39) $$\sum_{i=1}^{n}\alpha_{i}(\xi ,\eta )K_{vi}(x_{dav}+\eta x_{dbv})=(K_{0av}+\xi K_{0bv})[(x_{0av}+\xi x_{0bv})-(x_{dav}+\eta x_{dbv})]$$

where $x_{0av}$ is the mean part of the measured displacement vector $x_{0v}$, $x_{0bv}$ is the adjoint vector of $x_{0v}$ with respect to ξ. $K_{0av}$ and $x_{dav}$ are the mean parts of the stiffness matrix $K_{0v}$ and the measured displacement vector $x_{dv}$, respectively. $K_{0bv}$ is the adjoint matrix of $K_{0v}$ with respect to ξ, and $x_{dbv}$ is the adjoint vector of $x_{dv}$ with respect to η.

Then, following the procedure in Section 2.2, the stochastic damage index α can be acquired.

### 2.4. L_1_ Regularization Algorithm

In the process of damage identification, it is often encountered that the number of measured degrees of freedom is inconsistent with the number of damage indexes. The mentioned problem will cause the damage identification equations, including Equations (9), (12), (14) and (38), to become ill-posed equations. To solve this problem in damage identification and ensure the accuracy of the stochastic damage identification, the L_1_ regularization algorithm in the literature [42,43,44] is introduced to solve Equations (9), (12), (14) and (38).

Equation (39) can be simplified as the form of Lα=R, where L and R are all known matrices. Here $L=[K_{v1}(x_{dav}+\eta x_{dbv}),K_{v2}(x_{dav}+\eta x_{dbv}),\cdots ,K_{vn}(x_{dav}+\eta x_{dbv})]$, $R=(K_{0av}$$+\xi K_{0bv})[(x_{0av}+\xi x_{0bv})-(x_{dav}+\eta x_{dbv})]$. α can be solved by minimizing the objective function ‖Lα=R‖. To conduct a regularization strategy, the objective function is redefined as:

(40) $$J\left(\alpha \right)={\left\|L\alpha -R\right\|}_{2}^{2}+\tau {\left\|\alpha \right\|}_{1}^{1}$$

where τ is the regularization parameter, which satisfies τ>0. The regularization parameter can be determined by the L-curve criterion [42]. τ can control the trade-off between the sparsity of the solution and the amplitude of residual norm, where the term ${\left\|L\alpha -R\right\|}_{2}^{2}$ forces the residual to be small, and the term ${\left\|\alpha \right\|}_{1}^{1}$ enforces the sparsity of the solution.

### 2.5. Probability-Based Damage Identification

To describe the damage possibility of structures, the definition of failure probability in structural reliability [45,46] is introduced into the evaluation of damage probability. That is, the probability of structural damage at the element level can be defined as the probability that, for the *i*-th element, the stiffness parameter $k_{di}$ at damaged status is less than the intact stiffness parameter $k_{0i}$. Therefore, the probability of damage, $P_{d}^{i}$, of the *i*-th element in the structure can be written as:

(41) $$P_{d}^{i}=P(k_{0i}>k_{di})\left(i=1,2,\cdots ,n\right)$$

where the stiffness parameter $k_{di}$ and $k_{0i}$ are random scalars, such as the elastic modulus of a beam element. In accordance with Equation (41), the probability of damage of the *i*-th element can be calculated, using Equation (42), as:

(42) $$\begin{array}{l} P_{d}^{i}=\iint_{K_{di}<K_{0i}}f_{k_{d}}(k_{di})f_{k_{d}}(k_{0i})dk_{di}dk_{0i}\\ \;=\int_{0}^{+\infty }[\int_{0}^{K_{0i}}f_{k_{d}}(k_{di})dk_{di}]f_{k_{0}}(k_{0i})dk_{0i} \end{array}\;=\int_{0}^{+\infty }F_{k_{d}}(k_{0i})f_{k_{a}}(k_{0i})dk_{0i}$$

where $f_{k_{0}}(\cdot )$ and $f_{k_{d}}(\cdot )$ represent the probability density functions (PDFs) of the stiffness $k_{0i}$ and $k_{di}$, respectively. $F_{k_{d}}(\cdot )$ is the probability distribution function of the stiffness $k_{di}$.

In addition, the probability of damage can be equivalently determined by:

(43) $$P_{d}^{i}=P(\alpha_{i}<0)$$

From the process of the above derivation, it is found that no restriction is imposed on the probability distribution type of $k_{0i}$, $k_{di}$, and $\alpha_{i}$. Usually, the probability distribution of structural parameters in the initial model is assumed to be symmetric, so that the damage index of a structural element at intact status can be regarded as a random variable with zero mean [47]. Then, its corresponding damage probability $P_{d}^{i}$ is 0.5. When $P_{d}^{i}$ is less than 0.5, which indicates that the chance of occurrence of damage is low, one can regard the structural element as intact. Given that $P_{d}^{i}$ equals 1, the element is judged as absolutely damaged. Therefore, the damage probability of each element of the beam structure varies in the range from 0.5 to 1.0. To improve the sensitivity of damage identification, a new index, $\theta_{d}^{i}$, is presented and named the damage probability index, which is defined as ($P_{d}^{i}$ − 0.5)/0.5, and the value of $\theta_{d}^{i}$ is between 0 and 1. When the value of $\theta_{d}^{i}$ in the *i*-th element is close to 1.0, it means that the element is damaged. On the contrary, when $\theta_{d}^{i}$ approaches zero, the element keeps an intact state. According to a number of damage identification simulations, the threshold of the damage probability index is designed to be 0.5, which indicates that the possibility of damage is greater than the intact possibility.

**Figure 1:**
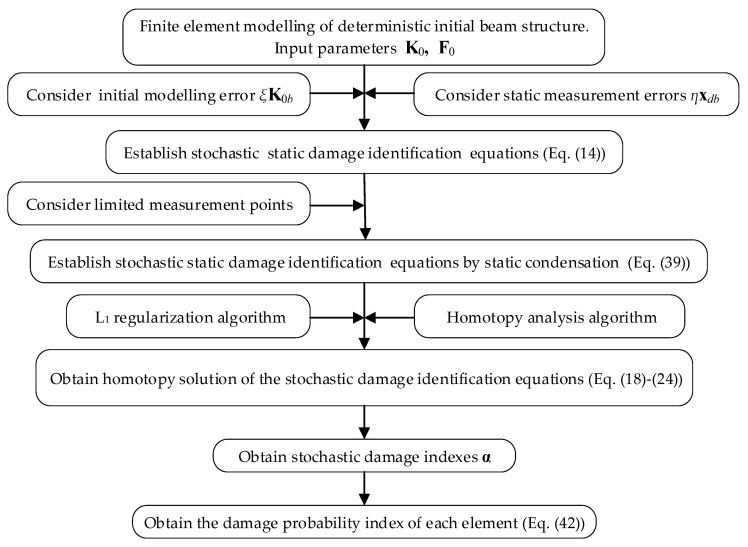
The flowchart of the proposed stochastic damage identification method.

To illustrate the steps of the proposed stochastic damage identification method, the flowchart of the method is plotted in Figure 1. The realization of this new method was implemented by the MATLAB software in this study. For practical structures, a large professional software package such as ANSYS would need to be used to implement the proposed method in the future.

## 3. Numerical Examples

To illustrate the proposed stochastic damage identification method, two numerical examples of beam structures are provided, including a simply supported beam and a continuous beam with various cross-sections. All cases in the examples assume that the randomness of beam structures is caused by the uncertainty of the elastic modulus, and both the modelling error and the measurement error are taken into account. In addition, it is proposed that the structural damage is caused by the reduction of the elastic modulus or bending rigidity of beam elements. As a matter of convenience, the proposed stochastic damage identification method is abbreviated as HDI. To demonstrate the accuracy of HDI, the direct Monte Carlo (MC) simulation method is used to provide a benchmark for the statistics of the stochastic damage indexes. To compare the HDI method, the first-order perturbation method (FPDI) is used for damage identification.

### 3.1. A Simply Supported Beam

**Figure 2:**
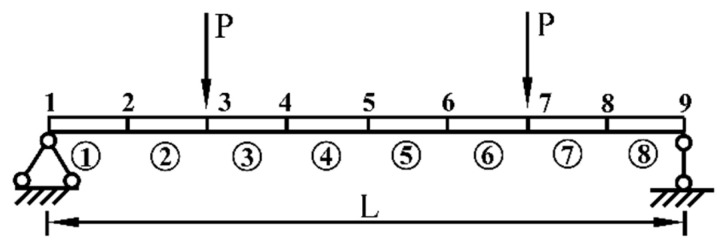
A simply supported beam structural model.

Consider a simply supported beam with a rectangular cross section, which is shown in Figure 2. The structural parameters are as follows: the length of beam **L** = 5 m, the cross-sectional area 0.15 m × 0.25 m, the moment of inertia 1.94 × 10^−4^ m^4^, and the elastic modulus 2.8 × 10^4^ MPa. The simply supported beam is divided into eight Euler–Bernoulli beam elements and nine nodes, and each node contains two degrees of freedom, including a vertical displacement and a rotational angle. It is assumed that at the initial status, the randomness of the stiffness of the simply supported beam is only caused by the uncertainty of the elastic modulus, and the elastic moduli of all elements are completely dependent, which are related to a stochastic variable of beta distribution. In static tests, two vertical concentrated loads **P** = 100 kN are applied at nodes 3 and 7, respectively, and only the vertical displacement of each node is measured. The rotational DOFs at all nodes are removed by the static condensation technique.

#### 3.1.1. Effect of Damage States

**Figure 3:**
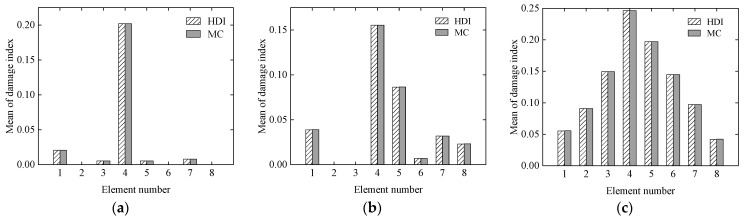
The mean of the damage index of each element under the three cases: (**a**) Case 1; (**b**) Case 2; (**c**) Case 3.

**Figure 4:**
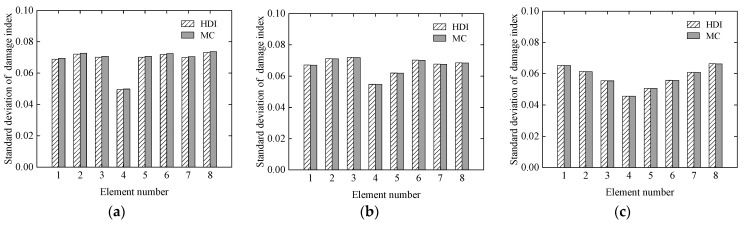
The standard deviation of the damage index of each element under the three cases: (**a**) Case 1; (**b**) Case 2; (**c**) Case 3.

To study the effectiveness of the proposed HDI method for identifying different damage locations and degrees, three various damage cases are listed in Table 1, where the reduction ratio of the elastic modulus is used to describe the damage degree of element. The reduction ratio of the elastic modulus of each element is defined as the relative error of the elastic modulus of the actual element to that of the same element at the initial mean status. It is assumed that the coefficient of variance (COV) of the elastic modulus for each element is 0.05 at the initial status, and the COVs of the static measurement errors of the beam after damaged are also 0.05. The static measurement errors are modelled as a beta distribution. The proposed HDI method and the MC method are used for damage identification. The identification results of the MC method with 1 × 10^5^ samples are used as a benchmark. The results of the damage identification using the two methods, which include the means and standard deviations (SD) of each element, are shown in Figure 3 and Figure 4, respectively.

From Figure 3 and Figure 4, it is derived that for the mean of damage index, the relative errors between the identified results by the HDI method and the MC method are about 0.1% under the three cases. In addition, for the standard deviation of damage index, the corresponding relative errors are less than 0.7%. These results indicate that identified results under the three cases using the HDI method are consistent with those of the MC method.

**Figure 5:**
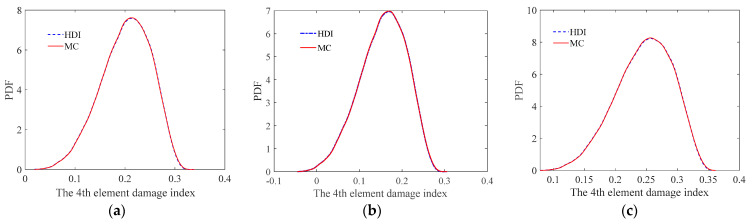
The PDFs of the damage index of the 4th element under the three cases: (**a**) Case 1; (**b**) Case 2; (**c**) Case 3.

To further illustrate the effectiveness of the HDI method, the PDFs of the damage index of the 4th element in the three cases are plotted in Figure 5. It is found from Figure 5 that the PDF curves of the damage index of the 4th element using the HDI method are close to those of the MC method.

**Figure 6:**
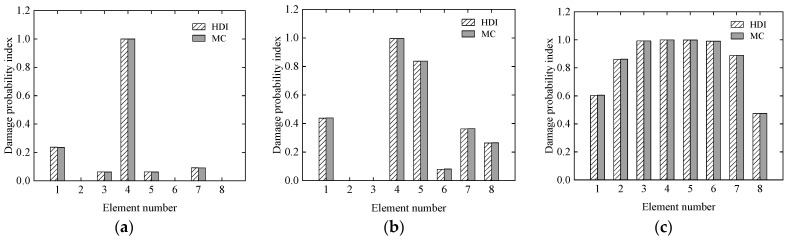
The damage probability index of each element under the three cases: (**a**) Case 1; (**b**) Case 2; (**c**) Case 3.

Finally, the damage probability indexes of all of the elements in the three cases are shown in Figure 6. From Figure 5, it is observed that the damage probability indices of all of the elements determined by the proposed method agree with the results of the MC method very well. It is also found from Figure 6 that the damage probability indexes of the 4th element under the three cases are about 1.0, which means that the occurrence of damage is almost absolute. With the exception of the 1st and 7th elements in Case 2, the assumed damage elements are confirmed by the corresponding damage probability indexes, which are more than 0.5, in all three cases.

It is worth mentioning that the proposed method can also be applied to other types of beam structures, and is not limited to the Euler–Bernoulli beam. In addition, to determine the damage indexes of structural elements, the damage identification equation has to be solved using all measured nodal displacements. Therefore, the nodal displacement cannot be regarded as a damage sensitive feature for the proposed method.

#### 3.1.2. Effect of Uncertainty of Measurement Errors

The effect of uncertainty of measurement errors on damage identification results of the simply supported beam was studied using the proposed HDI method and the MC method at different levels of uncertainties. For the uncertainties, the COVs of the measurement errors of the static displacements are assumed to be 0.1 and 0.15, respectively. The simulated damage situation is the same as that in Case 3 in Section 3.1.1. Both the measurement errors and the modelling error are assumed as to have beta distributions. The COV of the initial modelling error is still 0.05.

**Figure 7:**
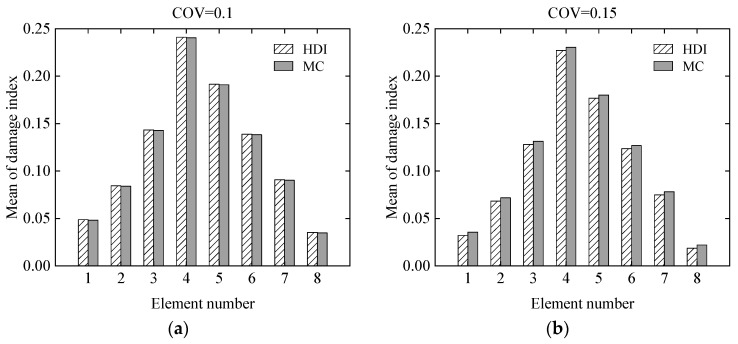
The mean of the damage index of each element at different uncertainties: (**a**) COV = 0.1; (**b**) COV = 0.15.

**Figure 8:**
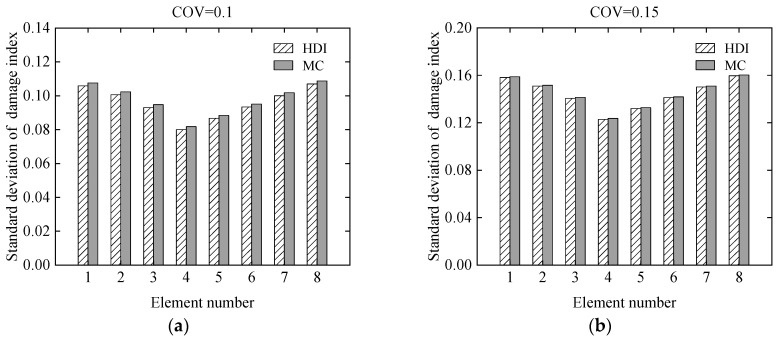
The standard deviation of the damage index of each element at different uncertainties: (**a**) COV = 0.1; (**b**) COV = 0.15.

**Figure 9:**
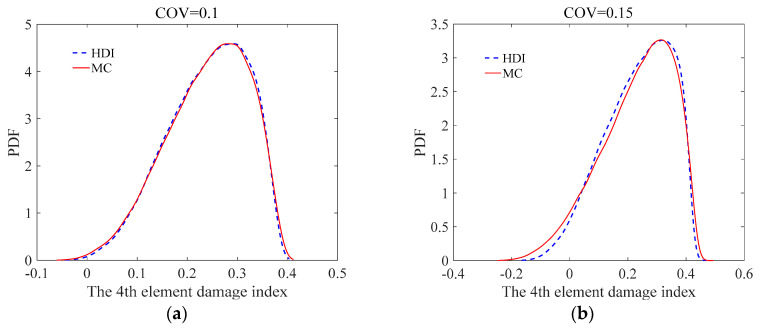
The PDF of the damage index of the 4th element at different uncertainties: (**a**) COV = 0.1; (**b**) COV = 0.15.

**Figure 10:**
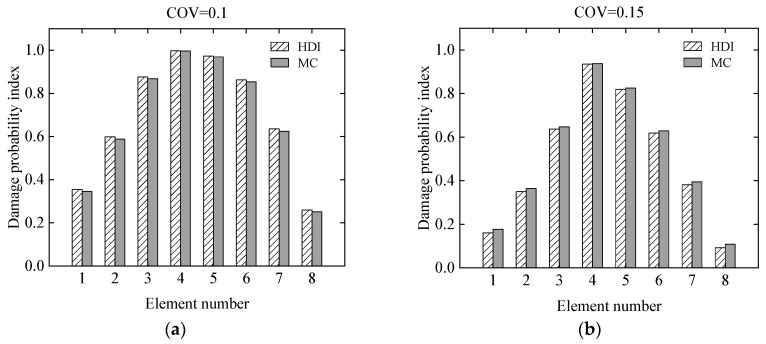
The damage probability index of each element at different uncertainties: (**a**) COV = 0.1; (**b**) COV = 0.15.

The mean and standard deviation of the damage index of each element computed by the proposed method are plotted in Figure 7 and Figure 8, respectively. From Figure 7 and Figure 8, it is found that the mean and standard deviation of the damage index of each element identified by the proposed HDI method coincide with those by the MC method. With the increment of the uncertainty of the measurement errors, the standard deviation or fluctuation of the damage index of each element will increase. The PDF of the damage index of the 4th element at different uncertainties is shown in Figure 9. Figure 9 shows that when the COV is 0.1, the PDF curve of the damage index of the 4th element using the proposed method is in a very good agreement with that of the MC method. When the COV reaches 0.15, the PDF curve of the damage index of the 4th element using the proposed method has little deviation from that of the MC method. The damage probability index of each element for different uncertainties is plotted in Figure 10. It is observed from Figure 10 that the results of both methods are almost the same, and when the COV becomes large, the damage probability index of each element will decrease. When the COV is 0.1, the 1st and 8th elements cannot be identified as damaged because the related damage probability indexes are less than 0.5. Correspondingly, due to the small damage probability indexes, it is impossible to determine the assumed damage in the 1st, 2nd, 7th, and 8th elements in the case that the COV is 0.15.

#### 3.1.3. Effect of Uncertainty of Modelling Error

**Figure 11:**
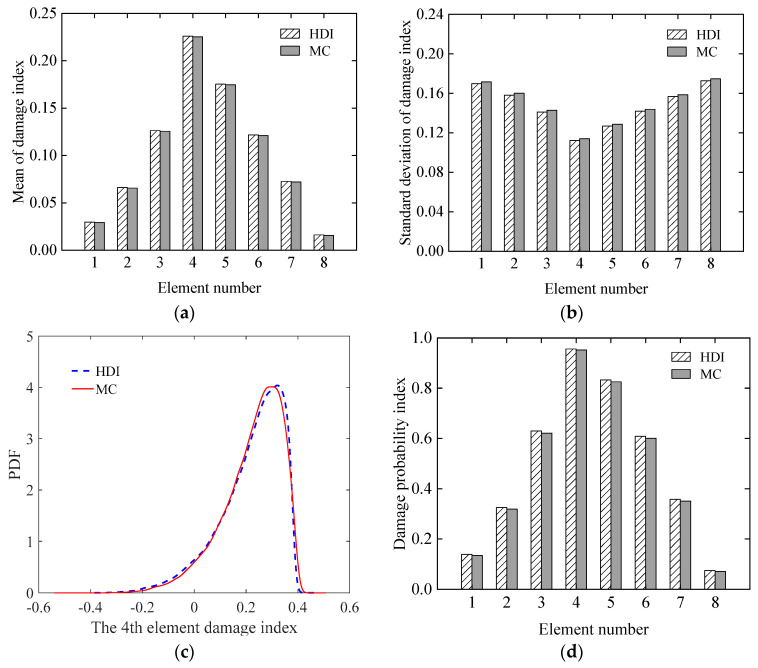
The statistics of the damage index of each element in the simply supported beam: (**a**) mean; (**b**) standard deviation; (**c**) PDF; (**d**) damage probability index.

The effectiveness of identifying damage of the proposed method when the COV of the modelling error increases from 0.05 to 0.15 is studied in this section. The assumed damage case is the same as that in Case 3 in Section 3.1.1. The COV of the measurement errors is assumed as 0.05. Using the proposed HDI method and the MC method, the mean and standard deviation of the damage index of each element, the PDF of the 4th element, and the damage probability index of each element are shown in Figure 11a–d, respectively. First, from Figure 11a–d, it is found that the results from the proposed method almost coincide with those using the MC method. When comparing the results in Figure 11a–d with those in Figure 3c, Figure 4c, Figure 5c and Figure 6c, where the COV of the modelling error is 0.05, respectively, it can be observed that when the COV of the modelling error increases, although the mean of the damage index of each element does not change much, the standard deviation of the damage index increases significantly, and the maximum value changes from 0.065 to 0.172. The shape of the PDF of damage index changes from symmetric to asymmetric. In particular, the damage probability index declines too much so that the damage probability indices of the 1st, 2nd, 7th, and 8th elements are less than 0.5, from which one cannot assume that these elements are damaged as discussed in Case 3 in Section 3.1.1.

### 3.2. A Continuous Beam with Variable Cross-Section

**Figure 12:**
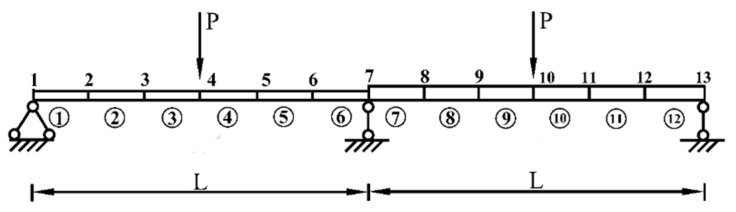
A continuous beam with a variable cross-section.

Consider a two-span continuous beam with variable cross-section as shown in Figure 12. It is supposed that the cross-sectional areas of the left and right segments in the beam are 0.15 m × 0.25 m and 0.15 m × 0.35 m, respectively. The cross section of the beam is rectangular. The length, **L**, of each span is 1.9 m. The elastic modulus is 2.8 × 10^4^ MPa. The continuous beam is divided into 12 identical beam elements and 13 nodes. Each node has two degrees of freedom, a vertical displacement, and a rotational angle. Two vertical concentrated loads, **P** = 100 kN, are applied to the mid of two spans, respectively. The COV of the modelling error of the continuous beam is assumed to be 0.05. The COVs of the measurement errors are 0.05, 0.1, and 0.15, respectively, indicating that the uncertainty of the measurement errors changes from small to large. To describe the damage degree of the beam element, the reduction ratio of the elastic modulus of each element is listed in Table 2. Three methods, the proposed HDI method, the FPDI method, and the MC method, are provided to implement the stochastic damage identification of a continuous beam with a variable cross-section.

**Figure 13:**
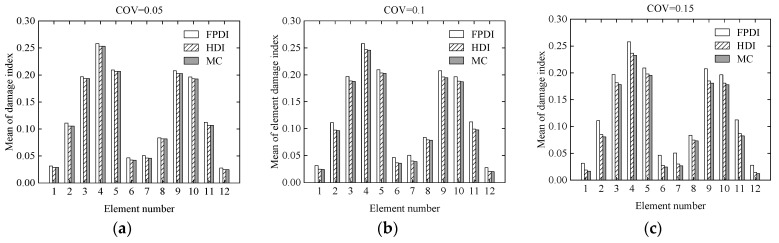
The mean of the damage index of each element for different uncertainties: (**a**) COV = 0.05; (**b**) COV = 0.1; (**c**) COV = 0.15.

**Figure 14:**
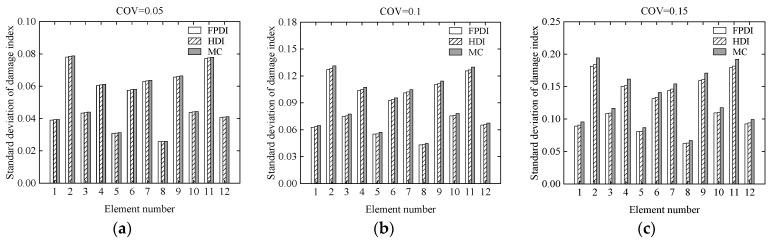
The standard deviation of the damage index of each element for different uncertainties: (**a**) COV = 0.05; (**b**) COV = 0.1; (**c**) COV = 0.15.

**Figure 15:**
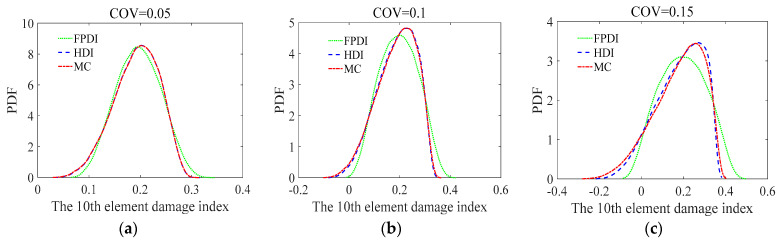
The PDF of the damage index of the 10th element for different uncertainties: (**a**) COV = 0.05; (**b**) COV = 0.1; (**c**) COV = 0.15.

**Figure 16:**
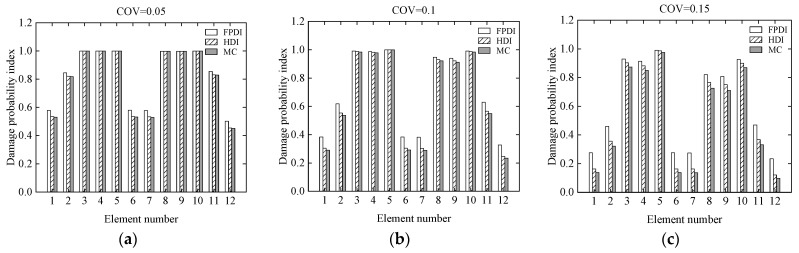
The damage probability index of each element at different uncertainties: (**a**) COV = 0.05; (**b**) COV = 0.1; (**c**) COV = 0.15.

Considering that the COVs of the measurement errors are 0.05, 0.1, and 0.15, respectively, the mean and standard deviation of the damage index of each element computed using the proposed method are plotted in Figure 13 and Figure 14, respectively. The PDF of the damage index of the 10th element for different uncertainties is shown in Figure 15. The damage probability index of each element at the three uncertainty levels is shown in Figure 16.

From Figure 13 and Figure 14, it is found that when the COV of the measurement error is 0.05 or the uncertainty of measurement errors is small, for both the mean and standard deviation of the damage index of each element, the results from the FPDI method agree very well with those from the suggested HDI method and are very close to the results obtained by the MC method. However, when the COV of the measurement error equals 0.15 or the measurement errors become relatively large, the means of damage index by the FPDI method gradually deviate from the results by the MC method compared with those from the HDI method. The more obvious difference between the FPDI method and the HDI method can be observed from the PDFs of the damage index of the 10th element as shown in Figure 15. It is seen that when the COV varies from 0.05 to 0.15, for the PDF of the damage index of the 10th element, the result of the FPDI method becomes different from that of the MC method, but the result of the HDI method is still very close to that of the MC method.

From Figure 16, it is found that when the COV of the measurement error varies from 0.05 to 0.1, the assumed small damages in the 1st, 6th, 7th, and 12th elements cannot be confirmed. When the COV reaches 0.15, even if the assumed damage degree in the 2nd and 11th elements is 10%, these elements cannot be judged as damaged because the related damage probability indexes are less than the threshold value of 0.5.

**Figure 17:**
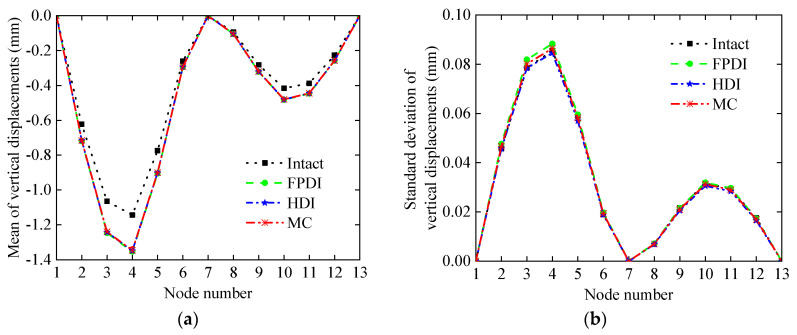
The statistics of the vertical displacement of each node in the continuous beam: (**a**) mean; (**b**) standard deviation.

Further, to directly show the influence of the vertical displacement on the damage of the continuous beam, the mean and standard deviation of nodal vertical displacement are plotted in Figure 17, where the COV of measurement errors is 0.05. As can be seen from Figure 17a, although the vertical displacement difference between the 6th and 8th nodes in the intact state and the damaged state is very small, the proposed method can still identify 5% of the damage degree in the 6th and 7th elements, according to the damage probability indexes of the 6th and 7th elements shown in Figure 16a, which are more than 0.5. The residuals between the vertical displacements of the 3th to 5th nodes at the intact and damaged states are also large and discernible because the assumed damage extent at that position is large.

**Figure 18:**
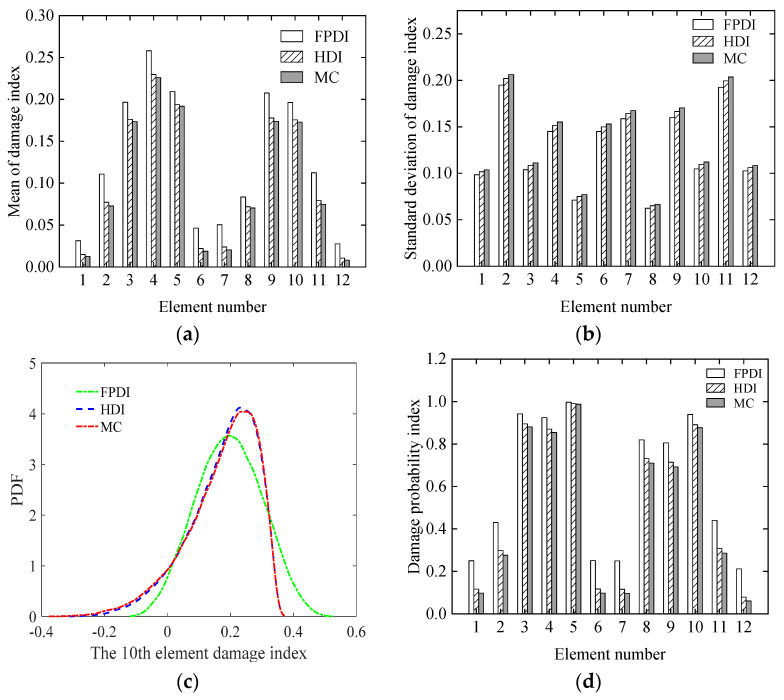
The statistics of damage index of each element in the continuous beam: (**a**) mean; (**b**) standard deviation; (**c**) PDF; (**d**) damage probability index.

Additionally, to illustrate the influence of the modelling error on the damage identification, assuming that the COV of the initial modelling error of the continuous beam is 0.15 and the COV of the measurement errors is 0.05, the statistics of the identified results using the HDI method and the FPDI method are plotted in Figure 18. From Figure 18a,c, it is found that the mean of the damage index of each element and the PDF of the damage index of the 10th element using the HDI method are approaching to those of the MC method more closely compared with those of the FPDI method. This finding also demonstrates that the HDI method has higher accuracy than the FPDI method in the case that the initial modelling error is relatively large.

In addition, it is found from Figure 18d that compared with the results in Figure 16a, where the measurement error is 0.05 and relatively small, the damage probability indexes in the 1st, 2nd, 6th, and 7th elements reduce significantly and are less than 0.5. These findings show that, in addition to the increase in the initial modelling error, the possibility of identifying damage decreases.

To study the computational cost of the proposed HDI method, for this modelling error situation, the computational time of the HDI method, the FPDI method, and the MC method are listed in Table 3. Although the FPDI method is only suitable for the damage identification problems with small uncertainty, it is generally considered to be an efficient method. It can be seen from Table 3 that the time consumption of the FPDI method is 443 s, which is about one-tenth that of the MC method. However, although the calculation time of the HDI method is 90 s longer than that of the FPDI method, the accuracy of the proposed method is significantly higher than that of the FPDI method, as shown in Figure 18. In addition, it can be seen from Table 3 that the calculation efficiency of the HDI method is much higher than that of the MC method.

## 4. Experimental Verification

**Figure 19:**
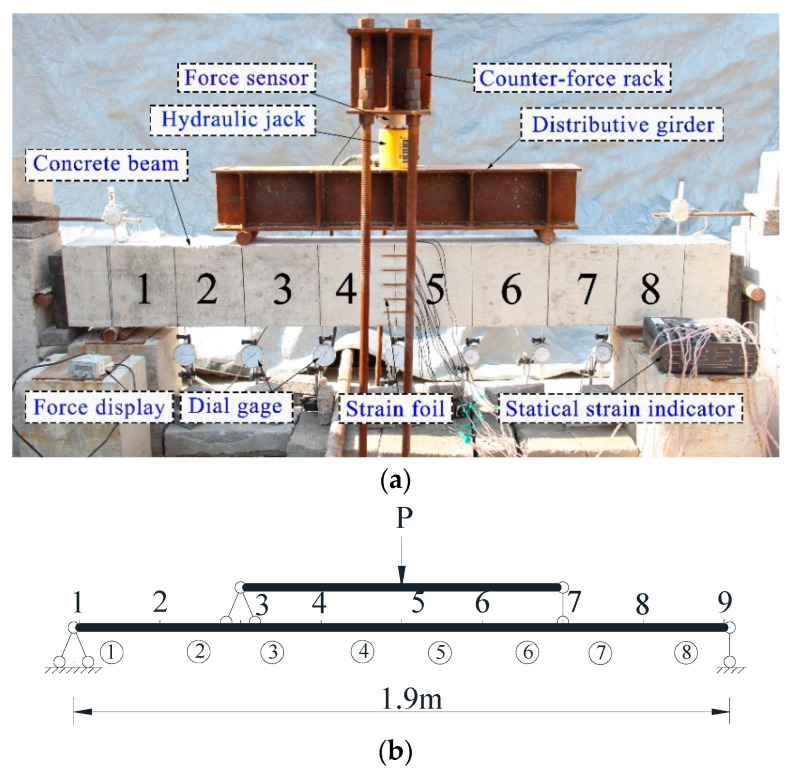
The static loading test of a simply supported concrete beam: (**a**) loading test; (**b**) mechanical model of the simply supported beam.

As described in this section, a series of static loading tests of a simply supported concrete beam, which is shown in Figure 19, were conducted to testify the effectiveness of the proposed identification method. These experimental tests comply with the test standard in the literature [48]. For the testing beam as shown in Figure 16, the length was 2200 mm, the span was 1900 mm, and the cross-sectional area is 150 × 250 mm^2^. Using a number of strength tests of the concrete used in the construction, one can determine the mean value of the elastic modulus as 2.8 × 10^4^ MPa. In the loading tests, only vertical deflections of the beam were measured. The mechanical model of the beam is shown in Figure 19b, where the simply supported beam is divided into eight Euler–Bernoulli elements with nine nodes. When the applied load **P** equaled 32 kN, the vertical deflections at seven nodes were measured several times using the dial gauges, and the measured vertical deflections and their statistics are listed in Table 4. Here it is supposed that all of the measured deflections are completely dependent and are assumed to have a beta distribution. The deflections of the tested beam can be measured using the vision-based measurement technique as a novel measurement mean [49,50]. Based on the actually measured concrete strength values, the COV of the elastic modulus in the initial beam element was assumed to be 0.1.

**Figure 20:**
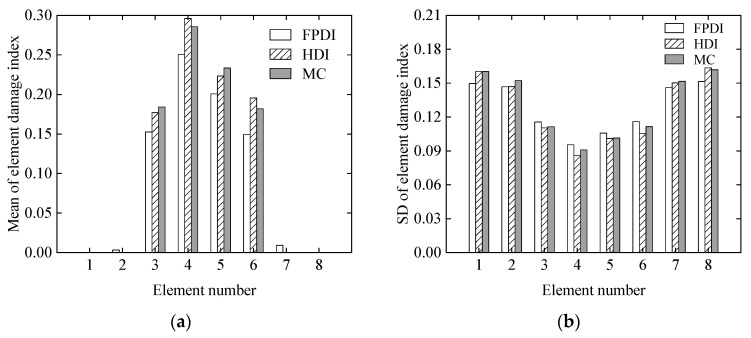
The mean and standard deviation of the damage index of each element with three different methods: (**a**) mean; (**b**) standard deviation.

Using the proposed HDI method, the FPDI method, and the MC method, the mean and standard deviation of the damage index of each element were calculated and are plotted in Figure 20. From Figure 20, it can be seen that the identified damaged elements are the 3rd, 4th, 5th, and 6th elements, which are located in the middle region of the simply supported beam. For the proposed method, the means of the damage indexes of the four elements range from 0.175 to 0.29, and their standard deviations range from 0.8 to 1.1. Because the means of the damage indices of the 1st, 2nd, 7th, and 8th elements are negative and near to zero, the damage situations of the 3rd, 4th, 5th, and 6th elements are discussed in the following paragraph. In addition, from Figure 20 it is found that the statistical results from the HDI method are closer to those of the MC method than that of the FPDI method, which indicates that the HDI method is better than the FPDI method in terms of computational accuracy.

**Figure 21:**
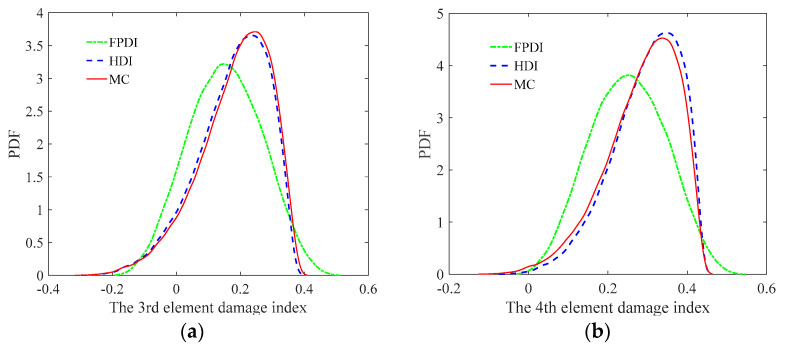

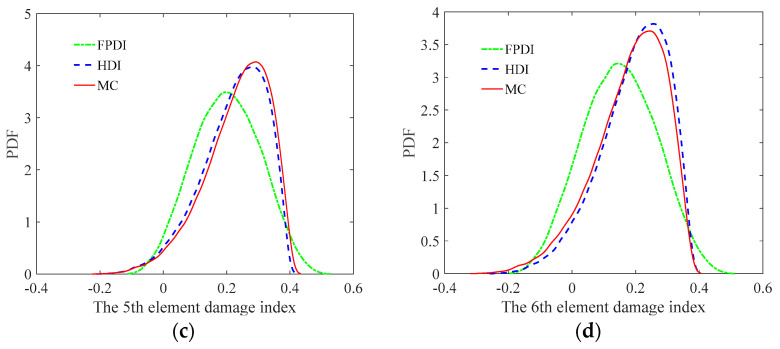
The PDFs of the damage indexes of the elements with three different methods: (**a**) the 3rd element; (**b**) the 4th element; (**c**) the 5th element; (**d**) the 6th element.

**Figure 22:**
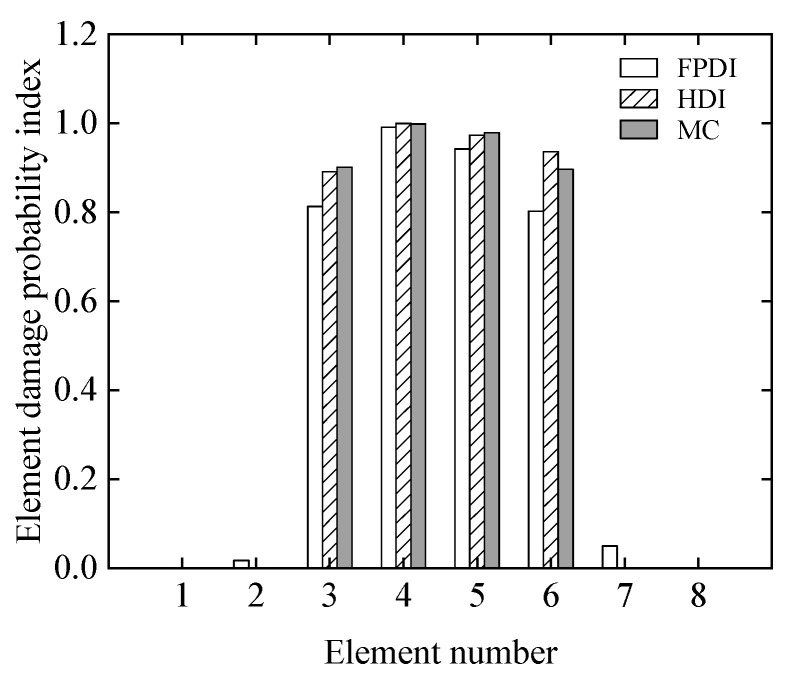
The damage probability index of each element with three different methods.

The PDFs of the damage indices of the 3rd, 4th, 5th, and 6th elements are shown in Figure 21, and the corresponding damage probability indexes are plotted in Figure 22. From Figure 21 and Figure 22, it is observed that the damage probability indexes of the four elements are large and far greater than 0.5. For the HDI method, the damage probability index of the 4th element is close to 1, and the damage probability indexes of other three elements vary from 0.86 to 0.95, which means that all four elements are definitely damaged. It is also obviously seen from Figure 21 and Figure 22 that through comparison with the identified damages of the MC method, the results from the HDI method have higher probability of damage than those using the FPDI method.

**Figure 23:**
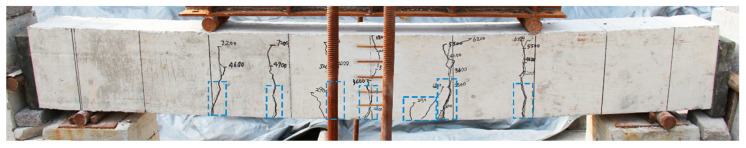
The cracks on the front of the concrete simply supported beam.

To check the identification effectiveness of the proposed HDI method, the crack status of the beam under different loads was recorded and is shown in Figure 23, where the marked numbers 4600, 4900, and 7200 indicate that the load **P** was 46, 49, and 72 kN, respectively. The cracks in the blue boxes occur when the load **P** is 32 kN. According to the literature [51] and the testing results, it was found that, overall, the beam is still located in an elastic or weak nonlinear status, which makes the proposed method available for damage identification. It can be found that the lengths of cracks along the height of beam are not significantly different, which means that the extent of damage of the beam locations corresponding to the 3rd, 4th, 5th, and 6th elements are almost the same. This phenomenon is consistent with the identified results using the proposed HDI method.

Similar to this experiment, relatively large uncertainty also exists in the measurement of real bridges, as illustrated in the reference [50]. It is expected that the proposed stochastic damage identification method could be used to identify the damage at the element or region level in an actual bridge in the future. In the damage identification of actual bridges, one still needs to solve problems related to sensor distribution, environmental conditions, etc.

## 5. Conclusions

This paper focuses on a novel damage identification approach of beam structures using uncertain static measurement data. This proposed new static damage identification method considers not only the measurement errors but also the initial modelling error, and regards them as random. The stochastic damage identification equations with respect to the damage indexes are established. A new homotopy analysis algorithm is used to solve the stochastic damage identification equations. During the process, a static condensation technique and the L1 regularization method are employed to deal with the limited measurement data and the ill-posed problems. Two numerical examples, a simply supported beam and a continuous beam, are provided to demonstrate the identification effectiveness of the proposed HDI method. Furthermore, a series of static tests of a simply supported concrete beam are implemented to verify the new stochastic identification method. The following conclusions can be drawn:

(1) The proposed HDI method is suitable for the structures with various damage degrees. However, if the uncertainty of the measurement errors and the modelling error is high, small damage will be suppressed and cannot be identified.

(2) In comparison to the FPDI method, the HDI method can ensure better accuracy in the damage identification of the beam with relatively large measurement errors and modelling error.

(3) On the premise of ensuring the accuracy of damage identification, the computational efficiency of the proposed HDI method is higher than that of the MC method.

(4) The identification results of the tested simply supported concrete beams using the HDI method are consistent with the phenomena observed in the static experiment.

## Figures and Tables

**Table 1:** Reduction ratio of the elastic modulus of each element under the three cases (%).

Case	1	2	3	4	5	6	7	8
Case 1	0	0	0	20	0	0	0	0
Case 2	5	0	0	15	10	0	5	0
Case 3	5	10	15	25	20	15	10	5

**Table 2:** The reduction ratio of the elastic modulus of elements in the continuous beam.

Element Number	1	2	3	4	5	6	7	8	9	10	11	12
Reduction ratio	5%	10%	20%	25%	20%	5%	5%	10%	20%	20%	10%	5%

**Table 3:** Computational time (s).

Method	HDI	FPDI	MC
CPU time	534	443	4158

**Table 4:** Measured vertical deflections and their statistic values in the case that **P** = 32 kN (mm).

No.	Number of Node						
	2	3	4	5	6	7	8
1	8.914	17.413	21.181	23.171	21.748	16.117	8.581
2	9.785	17.528	25.648	27.824	25.138	19.632	10.251
3	12.032	21.845	27.361	28.752	26.843	20.856	11.732
Mean	10.243	18.929	24.730	26.582	24.576	18.868	10.188
S.D	1.314	2.063	2.605	2.442	2.118	2.009	1.287
COV	0.128	0.109	0.105	0.092	0.086	0.107	0.126

## Data Availability

The data used to support the findings of this study are available from the corresponding author upon request.
